# Polysaccharides from *Spirulina platensis* (PSP): promising biostimulants for the green synthesis of silver nanoparticles and their potential application in the treatment of cancer tumors

**DOI:** 10.1186/s12934-023-02257-1

**Published:** 2023-12-05

**Authors:** Asmaa H. Al-Badwy, Ahmed M. Khalil, Ali H. Bashal, Rashad Kebeish

**Affiliations:** 1 Plant Biotechnology Laboratory (PBL), Botany and Microbiology Department, Faculty of Science, Zagazig University, Zagazig, 44519 Egypt; 2 Present Address: Biology Department, Faculty of Science Yanbu, Taibah University, 46423 Yanbu El-Bahr, Saudi Arabia; 3 Chemistry Department, Faculty of Science Yanbu, Taibah University, 46423 Yanbu El-Bahr, Saudi Arabia

**Keywords:** *Spirulina platensis*, Polysaccharides, Ag-NPs, FT-IR, TEM, Anticancer, Hep-G2, Apoptosis

## Abstract

Photosynthetic cyanobacterial components are gaining great economic importance as prospective low-cost biostimulants for the green synthesis of metal nanoparticles with valuable medical and industrial applications. The current study comprises the biological synthesis of silver nanoparticles (Ag-NPs) using soluble polysaccharides isolated from *Spirulina platensis* (PSP) as reducing and capping agents. FTIR spectra showed major functional groups of PSP and biogenic silver nanoparticles including O–H, C–H (CH2), C–H (CH_3_), C=O, amide, and COO– groups. The UV/Vis spectroscopy scan analyses of the extracted PSP showed absorption spectra in the range of 200–400 nm, whereas the biogenic Ag-NPs showed a maximum spectrum at 285 nm. Transmission electron microscopy (TEM) analysis of the synthesized Ag-NPs showed spherical nanoparticles with mean size between 12 and 15.3 nm. The extracted PSP and Ag-NPs exhibited effective cytotoxic activity against Hep-G2 (human hepatocellular carcinoma). The IC_50_ for PSP and Ag-NPs were 65.4 and 24.5 µg/mL, respectively. Moreover, cell apoptosis assays for PSP and Ag-NPs against the growth of Hep-G2 cells revealed superior growth inhibitory effects of the green synthesized Ag-NPs that encouraged tracing the apoptotic signalling pathway. In conclusion, the current study demonstrated an unprecedented approach for the green synthesis of silver nanoparticles (NPs), using the polysaccharide of *Spirulina platensis* as reducing and capping agents, with superior anticancer activity against a hepatocellular carcinoma cell line.

## Introduction

Algae are known to be one of the richest sources of protein, carotenoids, phycobiliproteins, phycocyanin, vitamins and sterols [1]. Numerous applications of algal polysaccharides in medicinal and biotechnological fields have been demonstrated [2]. Algal polysaccharides are stable, biodegradable, and extremely safe natural biopolymers. Thus, they play a significant role in delivery of drugs [3]. Commercially, they are applied in several industries as food, feed, beverages, emulsifiers, stabilizers, etc. [4]. Algal polysaccharides are regarded as a new generation of biopharmaceuticals because they have been effectively used as anticancer, antiviral, antioxidant, and anticoagulant compounds [5].

*Spirulina* is a genus of nutritious prokaryotes. It contains about 6–12% polysaccharide, 60–70% protein, in addition to fatty acids, vitamins, and minerals [6]. *Spirulina platensis* is therefore regarded as the most promising and nutrient-dense dietary source. Moreover, *Spirulina* displays biological functions in human body and plays an important role in medical and healthcare fields [7]. Polysaccharides of *Spirulina platensis* (PSP) have anti-inflammatory, anti-radiation, anti-fatigue, anti-mutation, antioxidation, antiaging, immune regulation, antitumor, and antiviral activities [8]. Algal polysaccharides have been widely employed as topical antioxidants in the cosmetics industry, notably in creams, lotions, and hygroscopic agents [9]. The synthesis of nanoparticles is gaining great interest to researchers all over the world because of their therapeutic effectiveness against infectious and non-infectious diseases, as well as their wide range of applications in industries like food, medicines, cosmetics, chemicals, agriculture, and pharmaceuticals. Algae, fungi, bacteria, plants, and their metabolites can all be utilized as reducing and stabilizing agents for the biogenic synthesis of nanoparticles [10]. Metal nanoparticles are very attractive field of research due to their cost-effectiveness and promising applications in various sectors [11]. Due to the biological properties of silver nanoparticles (Ag-NPs), they appear to be useful for biomedical applications, particularly in therapeutic interventions [12]. Ag-NPs are frequently used in pharmaceutical industries because they are stable at various temperatures range and have very low toxicity to human cells [13]. Ag-NPs are known to have promising biological activities including anticancer, antimicrobial, antiviral, antioxidant, antifungal, anti-inflammatory, and anti-angiogenic activities. Various biological based protocols, including the utilization of algal extracts, are thought to be safe, non-toxic, and giving a more ecologically friendly way to synthesize nanoparticles. The green synthesis of Ag-NPs mediated by microbes, plants and/or algal extracts is gaining great attention due to its sustainability, ease of handling, and cost-effectiveness compared to other methods [10]. Although cyanobacteria are considered as an ideal biological system for synthesis of noble metal nanoparticles, there are few reports about the usage of cyanobacteria in their synthesis [14]. Silver nanoparticles synthesized using algal extracts exhibit hydrophilic surface groups such as hydroxyl, carboxyl, and sulphate that give them special applicability because algae don’t produce any hazardous or toxic substances. Therefore, they can be used in medical treatments [15].

In the current study, polysaccharides of *Spirulina platensis* (PSP) were used for the green synthesis of Ag-NPs. The synthesized nanoparticles were physicochemically characterized and tested for their anticancer activity against Hep-G2 cell line in a direct comparison with the polysaccharides isolated from *Spirulina platensis*.

## Materials and methods

### Growth and maintenance of algal culture

*Spirulina platensis* was obtained from Phycology Lab, Faculty of Science, Zagazig University. *S. platensis* was cultured as previously described [16]. The algal isolate was cultured in Erlenmeyer flasks containing autoclaved Zarrouk’s medium. The pH of all media was adjusted to 9. The culture was incubated under a light intensity of 85–100 µEn^−2^ s^−1^ and light/dark cycles of 16 h/8 h. Algal cells were harvested by centrifugation at mid-logarithmic phase, washed three times with distilled water, and air-dried.

### Extraction, purification, and analysis of *Spirulina platensis* soluble polysaccharides

Soluble polysaccharides was extracted from dried cells of *Spirulina platensis* by hot water as previously described [17]. The resulting extract was centrifuged and the residues underwent three further extractions. Soluble polysaccharides were precipitated using 10% trichloroacetic acid (TCA) for 12 h at 4 °C. The precipitate was then collected using cooling centrifuge (MIKRO 200R Hettich zentrifugen, Germany). TCA was eliminated from the crude polysaccharide fraction by dialysis against distilled water followed by lyophilization. Soluble polysaccharides were further purified by DEAE-cellulose. The UV/VIS spectrophotometer (T80, PG Instruments Ltd., United Kingdom) was used to scan the highly purified sample in the 200–800 nm range to determine the absorption spectra of the isolated polysaccharides. Total carbohydrate contents were determined as previously described [18]. The method described by Lowry et al. [19] was applied to determine the total protein content of the extracted soluble polysaccharides.

### Synthesis of silver nanoparticles (Ag-NPs) via *Spirulina platensis* soluble polysaccharides

**Fig. 1:**
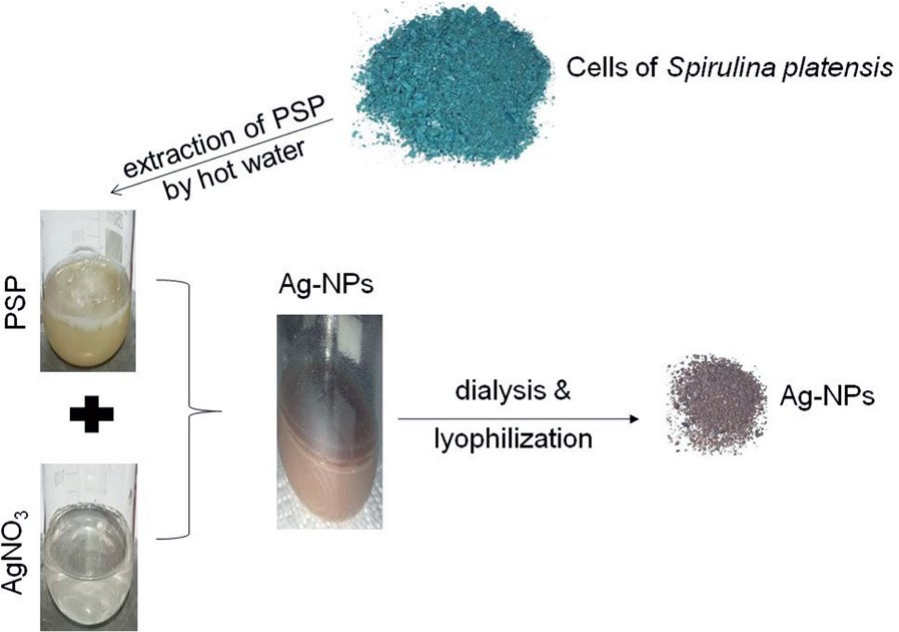
Sketch representing green synthesis of Ag-NPs by polysaccharides of *Spirulina platensis* (PSP)

Ag-NPs were synthesized as described by El-Rafie et al. [20] with some modifications. In brief, fifteen mg of *S. platensis* soluble polysaccharides were added to 50 ml distilled water. Three ml of 100 mM AgNO_3_ were dropped slowly to the polysaccharide solution with continuous stirring at room temperature (25 °C). The pH value of the reaction mixture was then adjusted to 10. The reaction volume was then completed to 100 mL by distilled water with continuous stirring at 70 °C for 20 min. The solution was then left in the dark for 24 h at room temperature followed by centrifugation for 5 min at 3000×*g*. The change in color of the solution to brown confirms the formation of Ag-NPs. The overall protocol of Ag-NPs synthesis is shown in Fig. 1.

### Characterization of the biosynthesized silver nanoparticles

#### Fourier‑transform infra red spectroscopy (FT‑IR)

To record the molecular functional vibration of chemical groups, present in PSP and biogenic Ag-NPs, Bruker FT-IR spectrometer was used (Nasdaq: BRKR). A mixture containing powdered potassium bromide (KBr), lyophilized polysaccharides and biogenic Ag-NPs pressed into pellets in the ratio of 1:100 were prepared and analyzed in Bruker FT-IR spectroscopy in the range of 500–4000 cm^−1^ at 4 cm^−1^ resolution [21].

#### UV–visible spectral analysis

The biogenic Ag-NPs synthesis has been firstly approved visually by the color change of the reaction mixes to brown (see Fig. 1). Green synthesized silver nanoparticles were then characterized using UV–Vis spectrophotometer (T80, PG Instruments Ltd. UK). Absorption spectrum of Ag-NPs in the range of 200–800 nm was recorded.

#### Transmission electron microscopy of Ag-NPs

Transmission Electron Microscope TEM (JEOL TEM-1400) operating at an accelerating voltage of 200 kV, at CURP Fact. of Agric., Cairo, Egypt, was used for determining the size and morphological properties of the biosynthesized Ag-NPs as previously described [22]. Samples for TEM analysis were prepared by drying the Ag-NPs under infrared lamp onto the carbon-coated copper grid before examination.

### Effect of the green synthesized Ag-NPs on Hep-G2 cell line

#### In vitro CPE assay

The antitumor properties of the soluble polysaccharide extracted from *S. platensis* and the biogenic Ag-NPs on Hep-G2 ATCC® HB-8065 cell line (Human hepatocellular carcinoma cell line) were evaluated by MTT-assay. In vitro, the cytotoxicity test was carried out following the procedure described by Mosmann et al. [23]. In brief, Hep-G2 cells were cultured into 96-well plates supplemented with 100 µL of culture medium in each well. Cells were incubated with dilution series of PSP as well as the biogenic Ag-NPs in DMSO (Sigma-Aldrich, Germany) to give a final concentration of 10, 25, 50, 75, 100, 200, 300, 500, 750, and 1000 µg/mL. A control containing only culture medium was applied. The assay was performed in triplicate. The cells were left in an incubator overnight to develop at 37 °C in a humidified 5% CO_2_ environment. After removing the growth medium, 100 µL of MTT working solution (0.4 mg/1 mL of PBS) were added. The supernatant was removed after 3 h of incubation and 100 µL of DMSO were then added to each sample. Then, absorbance at 550 nm was measured. The formula (Reading of extract/Reading of negative control) × 100, was applied to calculate the change in viability percentage. The IC_50_ value of PSP and Ag-NPs was also determined using CPE measurements of normal WISH cells (a human amnion-derived cell line). Based on a sigmoid concentration-response curve fitting models, the IC_50_ values were calculated using Graph Pad Prism computer software (International Scientific Community, San Diego, California, USA).

#### Cell apoptosis

Hep-G2 cell apoptosis assay was performed at Science Way center for research and consultations, Muqattam, Cairo, Egypt. Hep-G2 cells were pre-cultured in 25 cm^2^ flasks. The IC_50_ concentrations of PSP and Ag-NPs in RPMI-1640 and DMEM-media (Sigma-Aldrich, Germany) were then applied to the cells and incubated for 24 h. The cells were collected and fixed with 70% (v/v) ethanol in FBS (Sigma-Aldrich, Germany), then maintained overnight at 4 °C. The cells were then re-suspended in FBS containing 0.1 mg/mL RNase, 40 µg/mL penicillin, and 0.1% (v/v) Triton X-100 and incubated in a dark for 30 min at 37 °C. A flow-cytometer (Becton Dickinson, San Jose, CA, USA) supplemented with an argon ion laser at a wavelength of 488 nm were used to analyze the Hep-G2 cell apoptotic stages. The protocol described by Ozgur et al. [24] was applied to analyze the data using Multicycle Software (Phoenix Flow Systems, USA).

### Statistical analysis

Data are presented as the mean ± standard error of at least three independent measurements. Significance was determined according to student’s *t*-test using Excel software (Microsoft corporation, USA). Two-sided tests were applied for homoscedastic matrices.

## Results and discussion

### Extraction and characterization of *Spirulina platensis* soluble polysaccharides

Soluble polysaccharides have been isolated from dry *Spirulina platensis* powder following the hot-water extraction protocol as previously described [17]. Total protein content and total carbohydrate content were estimated after extraction. The observed total protein contents record 47.5 ± 2.2 mg g^−1^ and total carbohydrate contents record 325 ± 9.5 mg g^−1^ in the soluble polysaccharides fraction. The rules of carbohydrates in algae have been characterized. Carbohydrates act as endogenous reserve molecules for algal cell growth and are a structural component of their cell walls [25].

### Spectrophotometric analysis of extracted soluble polysaccharides from *Spirulina platensis*

**Fig. 2:**
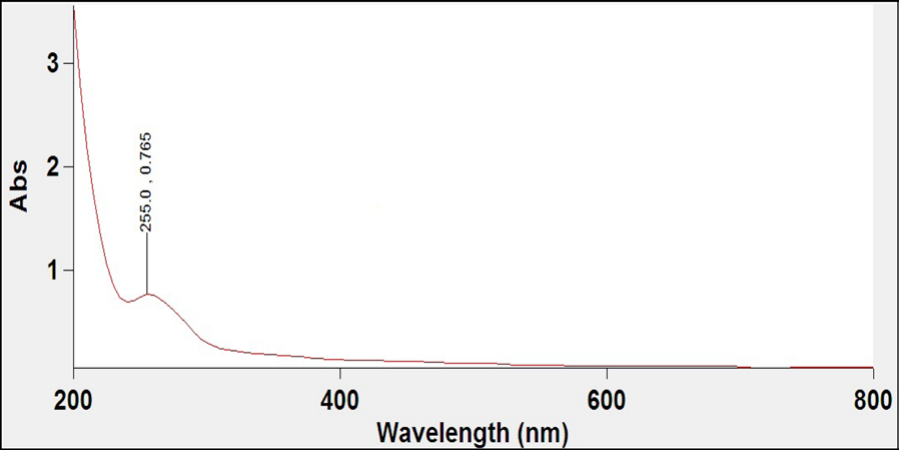
UV-absorbance spectrum of aqueous solution of soluble polysaccharide extracted from *Spirulina platensis*

UV spectrum is commonly applied for carbohydrate detection. Ultraviolet scan of soluble polysaccharides, isolated from *Spirulina platensis*, shows absorption spectra between 200 and 400 nm (Fig. 2). Jose et al. [26] demonstrated a prominent absorbance peak at 205–215 nm for sulfated polysaccharide isolated from *Padina tetrastromatica*. However, El-Naggar et al. [27] reported the presence of nucleic acids and proteins in the isolated polysaccharide fractions isolated from *Chlorella vulgaris.* This is confirmed by the presence of an absorption peak at 234 nm. The UV scan spectrum of polysaccharides isolated from *Spirulina platensis* in the current study revealed absorption peak in the range of 200–255 nm (Fig. 2) which is a characteristic of chemical groups; carboxyl, ester, carbonyl, and amine as previously reported [28]. The observed results of the spectrophotometric analysis of the extracted fraction of *Spirulina platensis* confirm therefore the presence of soluble polysaccharides.

### Fourier transform infra red (FT-IR) characterization of the extracted *Spirulina platensis* soluble polysaccharides

**Fig. 3:**
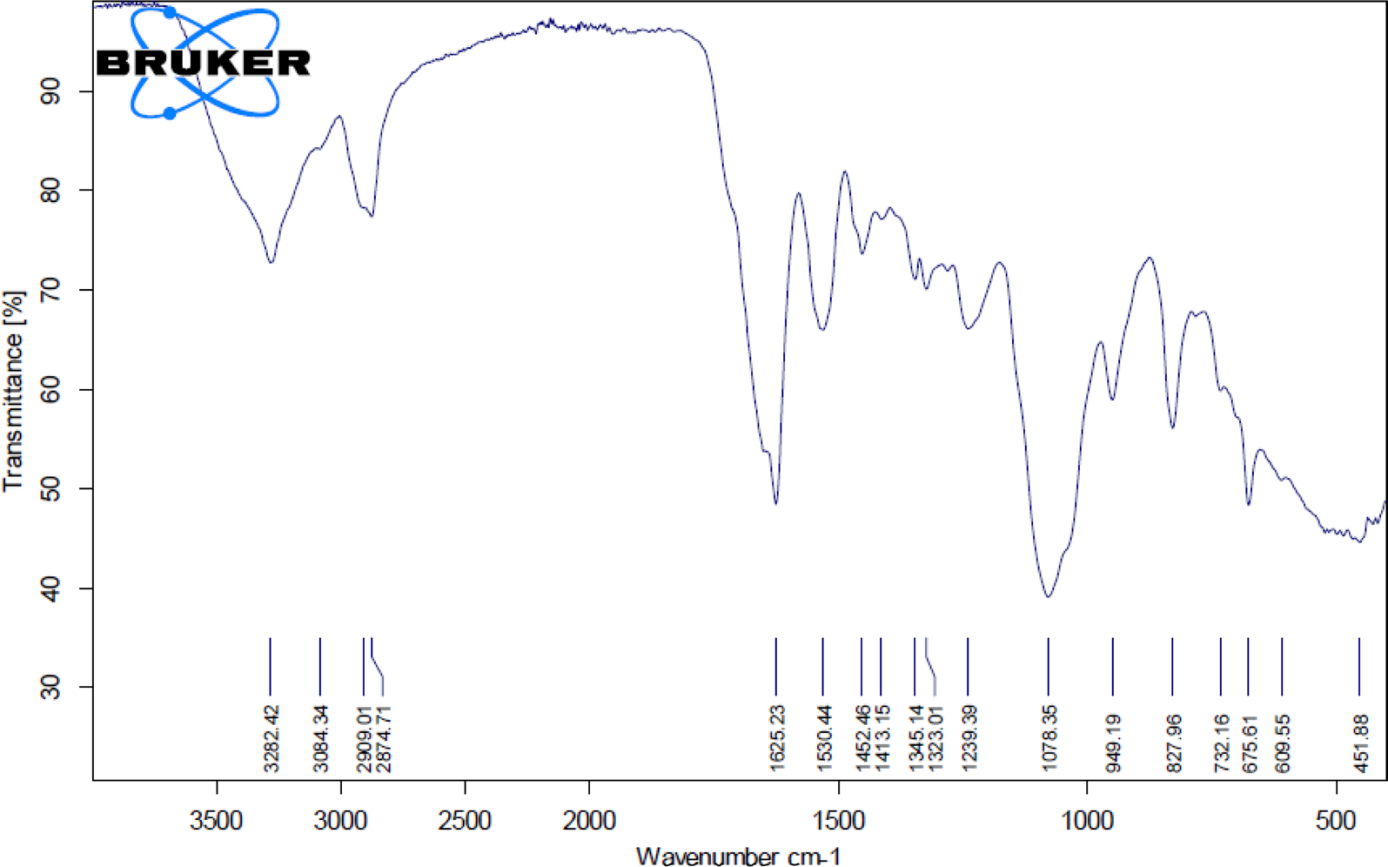
FT-IR characterization of *Spirulina platensis* soluble polysaccharides (PSP)

In order to identify the chemical functional groups, present in the components of *Spirulina platensis* soluble polysaccharides fraction (PSP), FT-IR analysis was applied (Fig. 3). FT-IR analysis of PSP revealed functional groups with peak frequencies of 3282, 3084, 2909, 2874, 1625, 1530, 1452, 1413, 1345, 1323, 1239, 1078, 949, 889, 827, 732, 675, 609, and 451 cm^−1^. These peaks referred to the presence of O–H, C–H (CH_2_), C–H (CH_3_), C=O, amide, and COO– groups (Table 1). The presence of a peak at 3282 cm^−1^ indicated the existence of O–H group. Peaks at 1452, 2874, 2909, and 3084 cm^−1^ are assigned to the presence of C–H stretched for (CH_2_) and C–H stretched for (CH_3_). However, FT-IR peaks recorded at 1413 and 1530 cm^−1^ represent the presence of C=O stretched for COO– and amide II functional groups (Table 1). FT-IR analysis of the biogenic Ag-NPs synthesized using PSP showed the absence of 3084, 2874, 1345, 889, 675, and 609 cm^−1^ peaks, see Table 1.

The band in the region 3282 cm^−1^ signifies the O–H stretch. The absorption bands in the range of 2920–2800 cm^−1^ are characteristics of C–H stretching vibration of CH_2_ or related to the secondary amines [29]. The FT-IR assay revealed the presence of a polysaccharide complex, as evidenced by the large absorptions around 3273 cm^−1^ and 2928 cm^−1^ that are corresponding to O–H stretching vibration and C–H stretching vibration of the –CH– groups, respectively [30]. Peng et al. [31] demonstrated that the absorption band in the region 2931 cm^−1^ suggests the stretching of CH_2_ group. Previous reports indicated that 1640 and 1320 cm^−1^ absorption peaks are related to the symmetric and a symmetric stretching vibration of the carboxyl group [32]. Beekes et al. [33] reported that the peaks between 1200 − 900 cm^−1^ are related to C–O–C, C–O dominated by ring vibrations of carbohydrates. Ajala et al. [34] demonstrated that sulfated polysaccharides from *Gelidium spinosum* was an acidic polysaccharide due to the presence of symmetric and a symmetric vibration of the carboxylate groups appeared at 1600 cm^−1^ and 1415 cm^−1^. Similarly, the absorption peaks at 1634, 1450 cm^−1^ represent the presence of uronic acid [35]. Moreover, the bands at 1030 and 1033 cm^−1^ are related to CO–C stretching as previously described [36]. Based on the observed results with FT-IR analysis, the extracted polysaccharides from *Spirulina platensis* (PSP) comprises the presence of O–H, C–H (CH_2_), C–H (CH_3_), C=O, amide, and COO– functional groups that play an important role in the green synthesis of Ag-NPs.

**Table 1:** FT-IR bands attributed to soluble polysaccharides and silver nanoparticles

(PSP) IR bands (cm^−1^)	Ag-NPs IR bands (cm^−1^)	Functional groups
3282	3282	O–H str
3084	Disappear	C–H str (sym) of –CH3
2909	2938	C–H str (asym) of > CH22909
2874	Disappear	C–H str (sym) of –CH3
1625	1632	Asymmetric and symmetric vibration of carboxylate
1530	1547	Amide II
1452	1467	CH def of > CH2
1413	1443	C=O str (sym) of COO
1345	Disappear	–
1323	1284	–
1239	1215	C–O–C, C–O dominated by ring vibrations of carbohydrates
1078	1093–1043	CO–C stretching
949	930	–
889	Disappear	–
827	801	–
732	784	CH rocking of > CH_2_
675	Disappear	Skeletal deformation bands
609	Disappear	–
451	447	–

### Green synthesis of Ag-NPs by *Spirulina platensis* soluble polysaccharides

*Spirulina platensis* soluble polysaccharides (PSP) were used for the biological synthesis of Ag-NPs. Upon incubation of the soluble polysaccharides’ solution of *Spirulina platensis* for 24 h with 100 mM silver nitrate in the dark, the color of the mixture turned brown. This indicates the reductive action of the extracted polysaccharides on silver nitrates to form Ag-NPs (Fig. 1).

#### UV characterization of the biogenic Ag-NPs

**Fig. 4:**
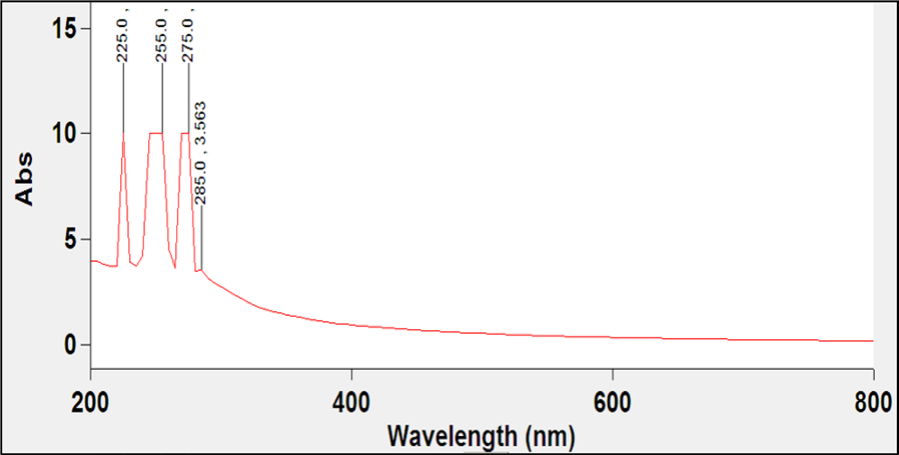
UV/VIS scan analysis of green synthesized silver nanoparticles

The synthesis of Ag-NPs was evaluated using an UV–VIS spectroscopy analysis. The increase in the intensity of the developed brown color is accompanied by gradual increase in the measured UV-absorbance during the synthesis process of silver nanoparticles. The absorption spectra revealed the presence of absorbance peaks at 225, 255, and 285 nm with absorption value at 285 nm of 3.563 corresponding to the presence of Ag-NPs (Fig. 4).

The highest color intensity of the synthesized Ag-NPs solution was recorded at the absorbance range between 200 and 400 nm. This is due to the formation of a large amounts of nanoparticles in response to the reduction power of the *Spirulina platensis* polysaccharides (PSP). The reaction mixture’s transformation to a brown color confirms the creation of silver nanoparticles. The observed UV analysis results are supported with the reported findings of El-Rafie et al. [20], since the color change of AgNO_3_ to brown confirms the formation of Ag-NPs. The absence of any peaks at 335 and 560 nm is an indication for the good stability and dispersion of the biosynthesized Ag-NPs [37].

Algal polysaccharides are reported to be involved in Ag-NPs synthesis. A report by El-Naggar et al. [38] approved the biosynthesis of Ag-NPs using soluble polysaccharides extracted from *Chlorella vulgaris*. Monomeric units of glucose, fructose, maltose, rhamnose, arabinose, and lactose make up the soluble polysaccharides. It has been reported that these polysaccharides have the ability to synthesize Ag-NPs when incubated with 100 mM AgNO_3_ for 24 h in the dark [39].

Algae are frequently used in the formation of nanoparticles. This is mostly because of their excellent ability to create nanoparticles on a large scale, inexpensive manufacturing costs, and their high capability to absorb metals and reduce metal ions. Algae’s superior capacity to withstand extreme air conditions compared to other microbes makes them an intriguing choice for the production of nanoparticles [40]. Both dry and fresh biomass of algae can be applied for the biosynthesis of nanoparticles, they are therefore considered as “Bionanofactories”. The length of time that necessary for the manufacture of silver nanoparticles is an additional benefit of employing algae. Algal-mediated synthesis of nanoparticles takes less time compared to other microorganisms. *E. coli* bacteria need to be incubated for 60 h before being used to produce Ag-NPs nanoparticles [41], whereas synthesis of Ag-NPs by *Caulerpa racemose* takes about 3 h [42]. Algae produce surface hydrophilic groups including carboxyl, hydroxyl, and sulphate that give silver nanoparticles a special range of applications. Since the probability of contamination with any toxic or hazardous substance is very rare, they can be used themselves in medical treatment [15].

#### FT-IR analysis of the biosynthesized Ag-NPs

**Fig. 5:**
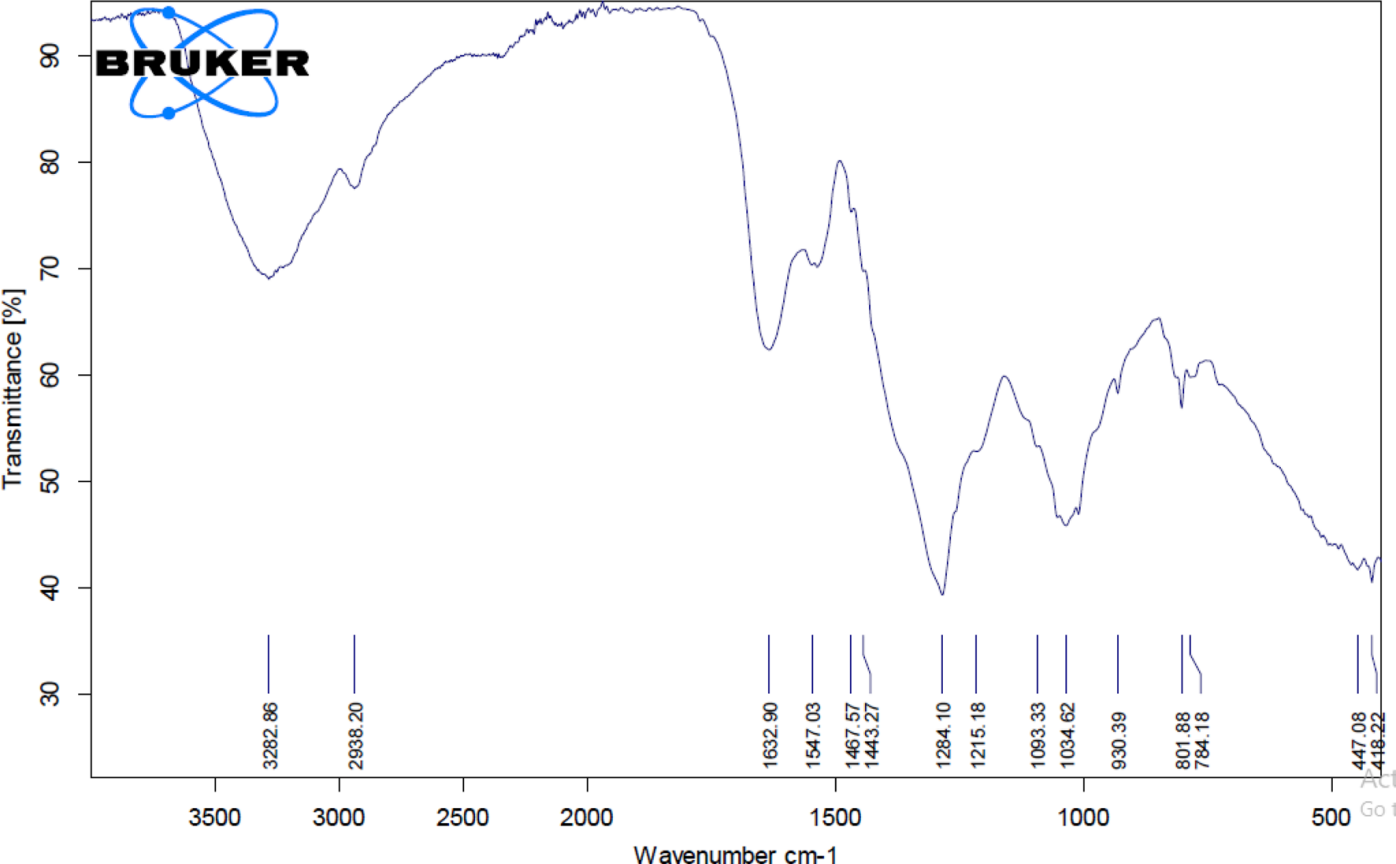
FT-IR spectrum of green synthesized silver nanoparticles

FT-IR analysis was also applied for characterizing the functional groups in the synthesized Ag-NPs (Fig. 5). Ag-NPs exhibit stretching of C–H groups, carbonyl groups, and amide protein groups, as shown by the distinctive functional group peaks at 3282, 2938, 1632, 1547, 1467, 1443, 1284, 1215, 1093, 1043, 930, 801, 784, 447, and 418 cm1 (Fig. 5). FT-IR spectroscopy helps to identify macromolecules responsible for the bio-reduction and formation of silver cations involved in the synthesis of the silver nanoparticles.

Previous studies revealed that the spectral peak at 1632 cm^−1^ is related to the vibrations of amide protein groups and algal polysaccharide carbonyl groups vibrations [20], whereas the peak at 2938 cm^−1^ is related to the stretching of the C–H group [43]. This indicates that proteins probably form a covering layer on Ag-NPs that prevents particle agglomeration and consequently lead to medium stabilization. Moreover, FT-IR data assigned to the presence of reducing sugars in the soluble polysaccharide’s solution. These reducing sugars have the ability to reduce silver atoms and consequently promote the synthesis of the nanoparticles [20]. FT-IR analysis of Ag-NPs synthesized by the freshwater green alga *Pithophora oedogonia* showed certain peaks corresponding to the existence of long chain fatty acids, terpenoids, and secondary amide derivatives which covered and stabilized Ag-NPs [44]. Therefore, it can be concluded that FT-IR analysis of Ag-NPs observed in the current study is in accordance with the observed results from the above-mentioned reports. The physical characteristics of the biosynthesized Ag-NPs using PSP were further characterized using Transmission Electron Microscopy (TEM).

#### Transmission electron microscopy of green synthesized silver nanoparticles

**Fig. 6:**
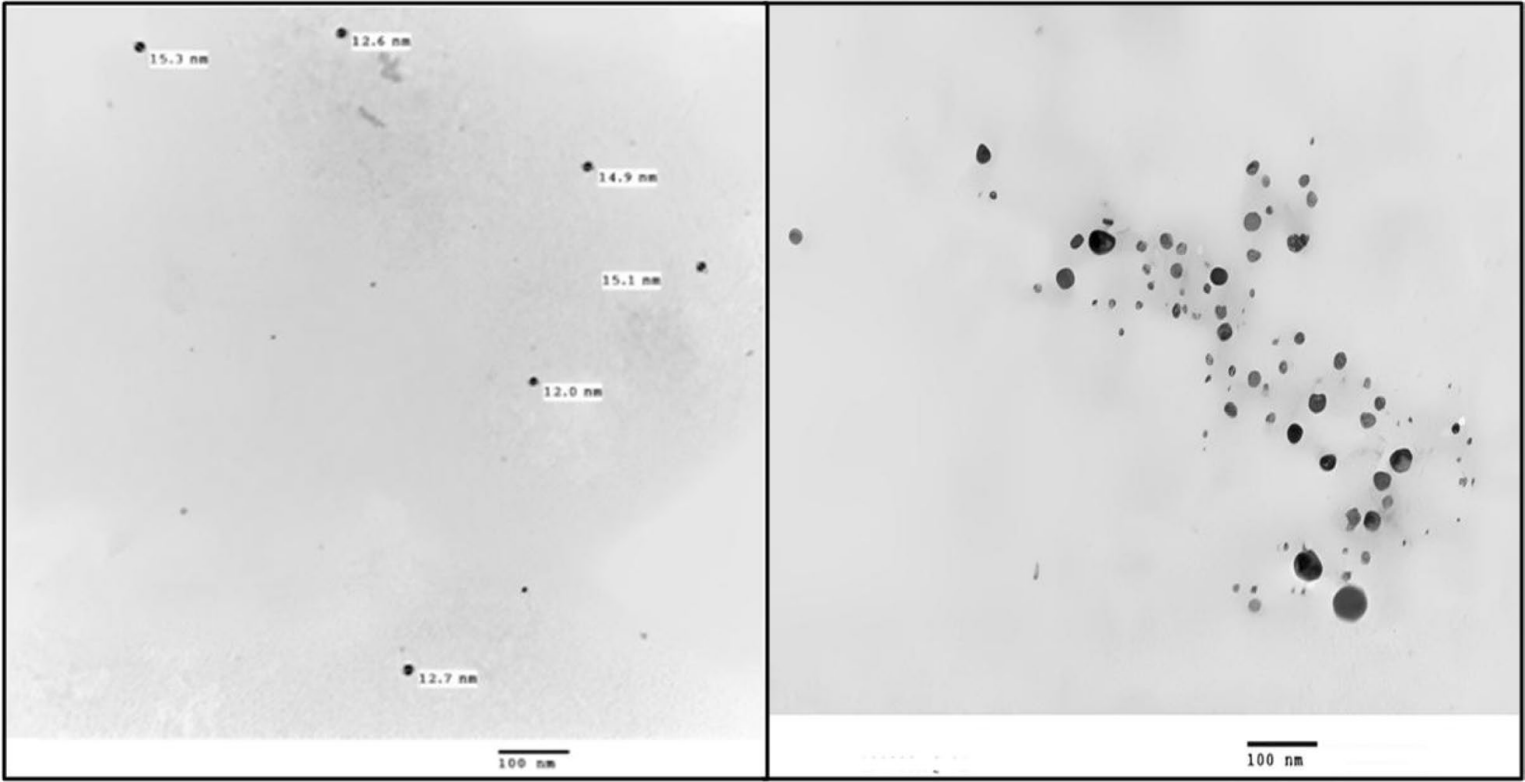
TEM micrographs illustrate the size and morphology of Ag-NPs obtained with polysaccharide extract at 100 nm

The biosynthesized silver nanoparticles via *Spirulina platensis* polysaccharide extract were also examined with TEM to confirm the formation of the biogenic Ag-NPs and to estimate their shape, aggregation, and particles size. As shown in Fig. 6, TEM was performed at 100 nm. Ag-NPs appear spherical in shape and most nanoforms are found in the size range of 12 to 15.3 nm. According to Mahdieh et al. [45], Ag-NPs synthesized extracellularly by *Spirulina platensis* characterized by its spherical shape with a size of ~ 12 nm at 25 °C. El-Naggar et al. [27] reported that silver nanoparticles synthesized by *Chlorella vulgaris* polysaccharides comprise an average size of 5.76 nm and they are spherical in shape. Polysaccharides are reported to be involved in Ag-NPs synthesis by algae. El-Rafie et al. [20] indicated that the synthesized silver nanoparticles using polysaccharides from *Colpmenia sinusa*, *Pterocladia capillacae*, and *Ulva faciata* were polydisperse and spherical in shape with maximum diameter size of 20, 12, and 7 nm; respectively. The observed TEM results indicate that *Spirulina platensis* soluble polysaccharides can form spherical shaped Ag-NPs with a diameter size range of 12 to 15.3 nm (Fig. 6).

### Antitumor activity of *Spirulina platensis* soluble polysaccharides and biogenic Ag-NPs

As anticancer drugs, Ag-NPs are widely used in the treatment of cancer because silver nanoparticles are more hazardous to malignant cells than other materials. They are frequently utilized as anticancer medications. Ag-NPs inhibit the growth of tumor cells due to their inhibitory effects in numerous signaling cascades that are important for the etiology and progression of cancer, with a minor fatal effect on normal cells [46]. A variety of human cancer cell lines, including IMR-90 lung fibroblasts, U251 glioblastoma cells, MDA-MB-231 and MCF-7 breast cancer cells, and endothelium cells, have shown promising anticancer effects of variable metal nanoparticles in recent years [47, 48].

#### Antitumor effect of soluble polysaccharides and Ag-NPs against Hep-G2 tumor cell line (CPE in vitro assay)

**Fig. 7:**
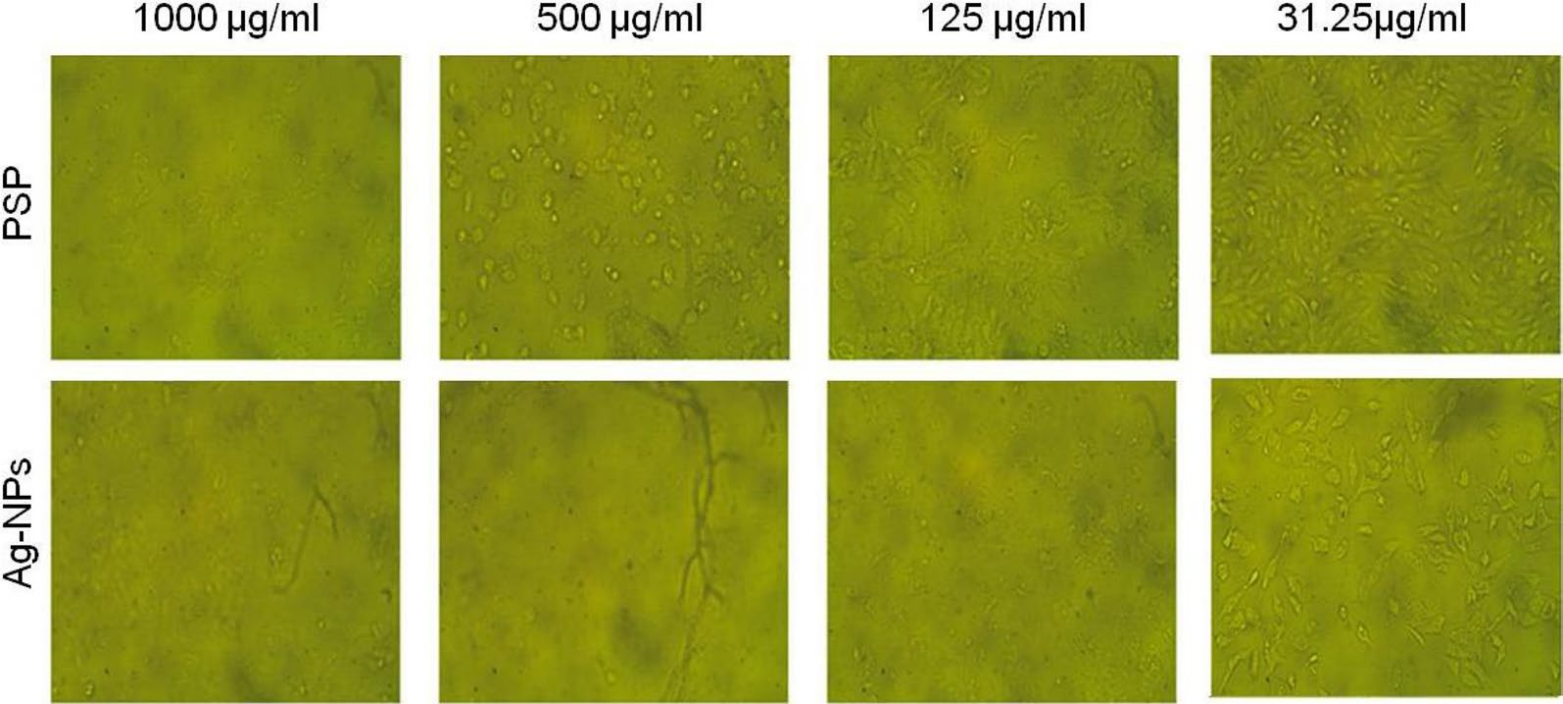
Morphological changes in Hep-G2 cells after PSP and Ag-NPs treatment

In vitro, the cytotoxic impact of both soluble polysaccharide extract from *Spirulina platensis* (PSP) and the green synthesized biogenic Ag-NPs was assessed against human hepatocellular carcinoma (Hep-G2) cell line and WISH normal cell line at varying concentrations. Results indicated a potent cytotoxicity against the tested tumor cell line (Fig. 7; Table 2). The occurrence of substantial cell death at high PSP and Ag-NPs concentrations is obvious (Fig. 7).

The extract of *S. platensis* soluble polysaccharides (PSP) and Ag-NPs showed significant anti-proliferative activity on the growth of Hep-G2 tumor cell line with superior actions of Ag-NPs. PSP and Ag-NPs at concentrations of 65.4 µg/mL and 24.5 µg/mL; respectively were able to inhibit cell proliferation at about 50% (IC_50_) after treatment. Moreover, the observed results showed that the effect of Ag-NPs is clearly more selective towards Hep-G2 cells compared to normal WISH cells line (Table 2).

**Table 2:** Cytotoxic activity of soluble polysaccharides and Ag-NPs on Hep-G2 cell line

Sample	IC_50_ (µg/mL)	
	Hep-G2	WISH
PSP	65.4 ± 1.26	112 ± 2.45
Ag-NPs	24.5 ± 0.53	043 ± 1.32

(*P*−value < 0.001)

Many studies have shown that algal polysaccharides can prevent the growth of different types of cancer cells in vitro [49]. Many polysaccharides characterized in marine algae such as calcium spirulan, carrageenan, and fucoidan etc., have been shown to have anti-tumor, anti-cancer, and anti-metastatic activities and significantly reduce cancer cell proliferation [50]. However, such activity is probably correlated with the presence of uronic and sulfate groups. Blue-green algae such as *Nostoc muscorum*, *Anabeana oryzae*, and *Calothrix marchica* showed high antiproliferative activity and high toxicity against Ehrlich ascites carcinoma (EAC) tumors [51]. Recently, promising anticancer effects of silver nanoparticles have been investigated in a variety of human cancer cell lines, including MDA-MB-231 and MCF-7 breast cancer cells, endothelium cells, IMR-90 lung fibroblasts, and U251 glioblastoma cells [47, 48]. The in vitro cytotoxicity of the biogenic Ag-NPs synthesized using *Oscillatoria limnetica* extract were evaluated against HCT-116, and MCF-7 with recorded IC_50_ values of 5.369 and 6.147 µg/mL, respectively [52]. Moreover, silver nanoparticles synthesized by *Desertifilum tharense* IPPAS B-1220, with a diameter size of 4.5–26 nm, were showed to have in vitro cytotoxicity against MCF-7, CaCo2, and Hep-G2 cancer cell lines with IC_50_ values of 58, 90, and 32 µg/mL, respectively [53].

The physiochemical interaction of silver nanoparticles with intracellular proteins and DNA may be the cause of their cytotoxic effects. Former studies have hypothesized that the anticancer activity of nanoparticles may due to the induction of apoptosis triggered by caspase 3 enzyme [54]. ROS production and JNK activation, mitochondrial depolarization, upregulation of caspase, calcium overload and death-inducing signals are all associated with apoptosis. Ag-NPs used as anticancer agents can be coated to alleviate its toxicity and increase their biological retention time, allowing a precise targeting of cancerous cells. In human breast adenocarcinoma cell lines, Ag-NPs from *Andrographis echioides* are frequently used because they have been shown to inhibit growth of MCF-2 cells. As the concentration of Ag-NPs rises, the viability of tumor cells decreased [55]. For tumor cells to continue growing, they need constant supply of nutrients and oxygen, which necessitates a dense network of blood vessels that is created through the angiogenesis process. According to reports, Ag-Nps inhibit the process of angiogenesis, which slows the growth of tumors [38]. The observed results of the cytotoxicity assay confirmed that the extracted *Spirulina platensis* polysaccharides and Ag-NPs are cytotoxic against Hep-G2 cells and the result of MTT assay in the current study is strongly in accordance with the previous findings.

#### Hep-G2 cell apoptosis

**Fig. 8:**
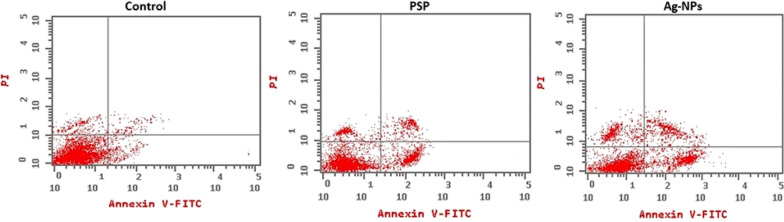
Effect of soluble polysaccharides and Ag-NPs on Hep-G2 cell apoptosis

The study was extended to evaluate the antitumor properties of the soluble polysaccharides isolated from *S. platensis* and the biogenic Ag-NPs through studying Hep-G2 cell apoptosis after applying the IC_50_ concentrations of the test sample. Untreated Hep-G2 cells were used as control. Results of Hep-G2 cell apoptosis assay are shown in Fig. 8; Table 3. Application of the IC_50_ concentrations of PSP extract and Ag-NPs to Hep-G2 cells results in significant reduction in normal cells (9.47% and 5.72% for PSP and Ag-NPs; respectively) and a great increase in necrotic cells (51.87% and 55.02% for PSP and Ag-NPs; respectively) compared to untreated Hep-G2 cells (1.68% necrosis). Biogenic Ag-NPs showed superior cell apoptosis effects compared to PSP under the assay conditions (Table 3).

**Table 3:** Effect of soluble polysaccharides and Ag-NPs on different stages of Hep-G2 cell apoptosis

Sample code	Normal cells (%)	Early apoptosis (%)	Late apoptosis (%)	Necrotic cells (%)
PSP	09.47	14.82	23.84	51.87
Ag-NPs	05.72	13.83	25.43	55.02
Control. Hep-G2	97.58	0.55	0.19	1.68

Cell apoptosis is widely considered to be one of the major parameters that incur growth loss to cancer cells. The apoptotic effect of polysaccharides in cancer cells was reported to be related to the production of reactive oxygen species (ROS), which lead to DNA damage and consequently cell death [56]. Polysaccharides have gained attention as possible chemical substances with strong anticancer properties against a range of cancer cell types. Additionally, polysaccharides can be created as substitutes for current cancer chemotherapeutic drugs because they have selective properties against tumor cells and little adverse side effects. It has been found that polysaccharides extracted from plants, fungi, microbes, and marine sources function primarily by inducing apoptosis on malignant cells [57]. Previous research have shown that *Sargassum fusiforme* polysaccharides can cause leukemia, gastric, bladder, and breast cancer cells to undergo apoptosis [58].

The biological functions of polysaccharides included both hydroxyl and carboxyl groups. Extracellular polysaccharides with carboxyl and hydroxyl groups have been shown in prior investigations to increase their anticancer and antioxidant properties [59]. The size of the nanoparticles, the Ag content, and the capping agents are the factors contributing to the increased cytotoxicity and reduced tumor cell growth. Due to their increased cellular absorption and extensive surface area for biomolecule interaction, small nanoparticles displayed higher toxicity [60].

El-Naggar et al. [38] reported that the size, concentration, and surface make-up of the nanoparticles are the main factors underlying their toxicity against cancer cells. Nanoparticles penetrate mammalian cells through phagocytosis or endocytosis. However, they additionally possess a surface charge that makes them more permeable to the matrix of cancer cells. The depth to which nanoparticles can penetrate cancer cells depends on their size because large nanoparticles can’t diffuse through the cancer matrix and can’t access the interior of the cancer cell [61]. Both Ag-NPs and Ag cations exhibit anticancer cytotoxicity through oxidative stress and inflammation, which leads to DNA damage and mitochondrial membrane potential disorder. Cytochrome-C is released, which leads to apoptosis and necrosis in the mitochondria that are related to cell proliferation and carcinogenesis [62].

The results observed in the current study showed an enhancement of early and late apoptotic cells percentage upon treatment (13.83%, 25.43% for Ag-NPs, and 14.82%, 23.84% for PSP), which encourages tracing the apoptotic signaling pathway. Taken together, the green synthesized Ag-NPs via *Spirulina platensis* soluble polysaccharides can induce cytotoxicity and cell apoptosis of Hep-G2 cells, therefore these nanoparticles could be effectively used as a promising cure material for the treatment of cancerous diseases.

## Conclusion

The current study comprises a promising approach for the green synthesis of silver nanoparticles via soluble polysaccharides isolated from *Spirulina platensis*. PSP was used as biostimulants for the synthesis of Ag-NPs with special physicochemical characteristics for therapeutic applications. Ag-NPs formation and characteristics were confirmed by UV–VIS spectroscopy, FTIR and TEM analyses. Both PSP and Ag-NPs have been shown to induce apoptosis in Hep-G2 cells. The green-synthesized Ag-NPs have stronger and superior growth inhibitory impact against Hep-G2 cells compared to PSP. The overall results strongly support the possible applications of the green synthesized Ag-NPs as a promising drug for the treatment of cancerous diseases. However, further investigations have to be performed to optimize the nanoparticles characteristics for their application in the medical fields.

## Data Availability

The authors confirm that all this study data is available within the article.
